# Supplementary material for: A Likelihood Approach for Real-Time Calibration of Stochastic Compartmental Epidemic Models
Source: PLoS Comput Biol. 2017 Jan 17;13(1):e1005257. doi: 10.1371/journal.pcbi.1005257 (PMC5240920; doi:10.1371/journal.pcbi.1005257)
Supplement: S1 File — (TAR.GZ) [file pcbi.1005257.s014.tar.gz › HSPH_Online-SI-Revision/IPoi09/n10-mild/n10-mild_BENCH2_summary.pdf]

peak -8	3w spec	3w cum	5w spec	5w cum	end	3w spec LOG	3w cum LOG	5w spec LOG	5w cum LOG	end LOG
mean	348.1	347.9	159.4	242.2	66.3	0.7	0.6	0.6	0.6	0.5
median	147.	184.8	86.3	138.8	53.1	0.5	0.5	0.5	0.4	0.3
quantile .1	86.2	90.2	45.3	64.4	27.8	0.3	0.3	0.2	0.3	0.2
quantile .9	912.5	865.4	411.7	621.8	116.6	1.2	1.2	1.	0.3	0.8
34	31	40	32	25						

peak -4	3w spec	3w cum	5w spec	5w cum	end	3w spec LOG	3w cum LOG	5w spec LOG	5w cum LOG	end LOG
mean	74.6	87.7	71.1	67.5	58.4	0.3	0.3	0.4	0.3	0.3
median	64.5	85.5	60.7	58.8	50.3	0.3	0.3	0.3	0.2	0.2
quantile .1	42.1	47.4	41.1	31.8	25.4	0.2	0.2	0.2	0.2	0.1
quantile .9	117.6	130.8	130.3	108.9	94.5	0.4	0.4	0.5	0.2	0.4
34	30	34	29	29						

peak	3w spec	3w cum	5w spec	5w cum	end	3w spec LOG	3w cum LOG	5w spec LOG	5w cum LOG	end LOG
mean	107.	97.8	139.7	100.1	106.7	0.3	0.3	0.4	0.3	0.3
median	81.4	91.8	82.9	80.1	75.5	0.3	0.3	0.3	0.2	0.3
quantile .1	48.2	44.5	46.7	45.5	38.4	0.2	0.2	0.2	0.2	0.2
quantile .9	214.5	163.4	245.1	181.6	233.3	0.5	0.4	0.6	0.2	0.5
25	21	28	20	28						

peak +4	3w spec	3w cum	5w spec	5w cum	end	3w spec LOG	3w cum LOG	5w spec LOG	5w cum LOG	end LOG
mean	167.4	124.	776.2	131.4	143.1	0.4	0.3	0.5	0.3	0.3
median	98.5	102.2	105.2	106.7	84.1	0.3	0.3	0.3	0.3	0.3
quantile .1	52.9	53.6	54.7	51.1	48.5	0.2	0.2	0.2	0.2	0.2
quantile .9	308.6	248.1	702.3	238.	316.7	0.6	0.5	0.7	0.2	0.6
35	31	34	33	33						

peak +8	3w spec	3w cum	5w spec	5w cum	end	3w spec LOG	3w cum LOG	5w spec LOG	5w cum LOG	end LOG
mean	764.7	865.1	1428.	987.8	1056.7	0.5	0.4	0.6	0.4	0.5
median	89.2	90.6	93.8	88.3	84.6	0.3	0.3	0.4	0.3	0.3
quantile .1	51.2	53.1	55.9	51.5	50.5	0.2	0.2	0.2	0.2	0.2
quantile .9	865.9	643.2	2729.7	653.1	757.4	0.9	0.8	1.3	0.2	0.8
35	33	37	34	34	{}	{}	{}	{}	{}	{}
